# Electrical Conduction Mechanisms in Ethyl Cellulose Films under DC and AC Electric Fields

**DOI:** 10.3390/polym16050628

**Published:** 2024-02-26

**Authors:** Jesús G. Puente-Córdova, Juan F. Luna-Martínez, Nasser Mohamed-Noriega, Isaac Y. Miranda-Valdez

**Affiliations:** 1 Facultad de Ingeniería Mecánica y Eléctrica, Universidad Autónoma de Nuevo León, Av. Universidad s/n, Cd. Universitaria, San Nicolás de los Garza 66455, Mexico; nasser.mohamednr@uanl.edu.mx; 2 Department of Applied Physics, Aalto University, P.O. Box 11000, FI-00076 Espoo, Finland; isaac.mirandavaldez@aalto.fi

**Keywords:** ethyl cellulose, electric conduction, fractional calculus, Schottky effect, AC conductivity

## Abstract

This work reports the dielectric behavior of the biopolymer ethyl cellulose (EC) observed from transient currents experiments under the action of a direct current (DC) electric field (~10^7^ V/m) under vacuum conditions. The viscoelastic response of the EC was evaluated using dynamic mechanical analysis (DMA), observing a mechanical relaxation related to glass transition of around ~402 K. Furthermore, we propose a mathematical framework that describes the transient current in EC using a fractional differential equation, whose solution involves the Mittag–Leffler function. The fractional order, between 0 and 1, is related to the energy dissipation rate and the molecular mobility of the polymer. Subsequently, the conduction mechanisms are considered, on the one hand, the phenomena that occur through the polymer–electrode interface and, on the other hand, those which manifest themselves in the bulk material. Finally, alternating current (AC) conductivity measurements above the glass transition temperature (~402 K) and in a frequency domain from 20 Hz to 2 MHz were carried out, observing electrical conduction described by the segmental movements of the polymeric chains. Its electrical properties also position EC as a potential candidate for electrical, electronics, and mechatronics applications.

## 1. Introduction

Ethyl cellulose (EC) is a biopolymer cellulose derivative whose main applications are in the pharmaceutical and food industries [1,2,3,4]. From the electrical point of view, EC has applications as a binder in printing pastes, electrets, and thermoelectric materials [5,6,7]. The macromolecular structure of EC consists of repetitive units of anhydroglucose. EC synthesis proceeds through the reaction between cellulose and ethyl chloride under alkaline conditions. The properties of the resulting EC will depend on the degree of substitution of the ethyl ether group. Among the diverse types of EC synthesized, the most used in industrial sectors is that with a degree of ethyl ether substitution between 2.4 and 2.5 [2,8]. This substitution degree provides important properties, for example, water resistance and solubility in polar and non-polar solvents, and certain azeotropic mixtures. The difference in size and electronic configuration of the atoms of EC induces an asymmetric spatial distribution of electric charge carriers, where each chemical group of the polymer chains is associated with an electric dipole moment. The molecular mobility of electric dipoles is of great interest for the characterization of the polymer structure, i.e., dielectric analysis and infrared spectroscopy.

The study of the electrical properties of organic polymeric materials is a fundamental research topic for the development of new devices and technologies. Polymeric materials exhibit two distinct electrical behaviors: they can store electric charge as a dielectric, and they can conduct electricity due to the movement of charge carriers. To fully understand the electrical properties of polymers, it is important to study the electric polarization and space charge phenomena that occur within them. By characterizing these phenomena, we can gain valuable insights into the behavior of polymeric materials in various electrical applications. The presence of space charge enhances the local electric field; in the absence of an external electric field, this electric field is different from zero, which seriously affects the dielectric properties of polymers and accelerates aging processes. Therefore, identifying the conditions and conduction mechanisms is fundamental to unraveling why they accumulate electrical charges on the polymer surface or as a bulk. In this sense, Khare et al. obtained thermograms by thermally stimulated discharge currents (TSDC) for EC, which consist principally of two relaxation peaks located at 333 K and 413 K. The first of these is attributed to the disorientation of ethoxy groups of the glycosidic units and the second to the diffusion of the space charge on the electrodes or to their thermal release from traps [9]. In addition, the authors performed current–voltage measurements, interpreting that the Schottky–Richardson mechanism dominates the electrical conduction process [10]. Bidault et al. [11] carried out dielectric spectroscopy measurements for EC, which exhibited three secondary relaxations at temperatures below its glass transition (~403 K). The comparison of the relaxation time and activation energy with other polymers allowed them to attribute the relaxation to the side groups and local movements of the main chain. Kumanek et al. [5] have reported the synthesis of multi-walled carbon nanotubes and the preparation of composite films with an ethyl cellulose matrix, which have potential applications in new energy technologies. They evaluated the thermoelectrical properties of the films, tuning the electrical response with heteroatoms, so that the material simultaneously demonstrated the highest possible value of electrical conductivity and Seebeck coefficient while restricting the thermal conductivity value to its minimum. More recently, Reyes-Melo et al. [2] fabricated and synthesized hybrid films with silver nanoparticles into an ethyl cellulose matrix. The authors studied the mechanical and dielectric relaxations of these films and found a remarkable interaction between the nanoparticles and the chemical groups of the organic matrix.

Although there are several reports in the literature about the electrical response of EC and its composites, there are still questions to be resolved regarding the space charge, charge transport, and the relationship between the structure and electrical properties of EC. This work evaluates the structure and morphology of EC using dynamic mechanical analysis and X-ray diffraction, and the measurement of transient currents at different DC electrical fields under vacuum conditions. Our goal is the application of fractional calculus for the analysis of the transient currents in polymers as an innovative tool. The analysis of the electrical conduction mechanisms of EC films is covered by several physical models in order to obtain an insight on the generation and transportation of carriers. Finally, AC conductivity at temperatures above their glass transition temperature was carried out. Experimentation and mathematical models were used in this study to understand the polymer’s electrical behavior, explain potential failures in service, and optimize its properties for insulator and dielectric applications.

## 2. Electrical Conduction in Polymers

Polymers that are free of chemical impurities and structural defects have no valence electrons that can exhibit an electronic current like in metals. According to the energy band theory, polymers typically have a bandgap energy higher than 5 eV but does not explain the origin of the electric currents produced, in the order of the picoamperes (pA). Therefore, to describe the conduction mechanisms of polymers at medium and high electric fields, the type and density of traps (potential wells) must be considered, in addition to the atomic structure. In this sense, several authors have modified the energy band theory to adapt it to the study of the electrical behavior of polymers [12,13,14]. One way to address conduction mechanisms in polymeric materials is through the experimental measurement of transient currents, monitoring the evolution of the electric current over time. Two main contributions make up the transient currents: the first is associated with the polarization current, which depends on the number of electric dipoles oriented in the direction of the electric field, and the second is related to a conduction current, which is assembled in two groups. The first corresponds to electric currents that are a function of the injection of electrons or holes in the polymer through the polymer–electrode interface (extrinsic charge carriers). The second group corresponds to electric currents associated with the movements of the electric charge carriers that are in the volume of the polymer (intrinsic charge carriers).

The conduction mechanisms controlled by the bulk are complex and have several origins. The orientation of electrical dipoles (permanent or induced) by the effect of an external electrical field produces a current that can be measured [15,16]. Additionally, the injection of electrons can be evaluated through an electric current. After the injection of electrons, these can be trapped in two ways, producing an accumulation of electrical charge in the polymer, considered as space charge. The mobility of the electric charge carriers is significantly restricted when they are trapped or occupy the localized energy states. The energy associated with trapping charge carriers also depends on the temperature, electric field, and energy depth of the traps. The first type of trapping is identified as physical or shallow traps and corresponds to changes in the conformational states of the polymer; the energy associated with this trapping mechanism is around 0.1–0.5 eV. The second trapping mechanism is identified as a chemical or deep trap, and is related to the formation of free radicals, chemical cross-linking, or breakage of covalent bonds; the energy associated with these traps has been reported as >1 eV. Several physical models are proposed in the literature for describing and understanding the nonlinear behavior and conduction mechanisms in polymeric materials: conduction mechanisms through the electrode–polymer interface (Schottky and Fowler–Nordheim) and those corresponding to the bulk (Poole–Frenkel, Space Charge Limited Current SCLC and Ionic-Hopping) [17,18,19].

Regarding the injection mechanisms through the electrode–polymer interface, the Schottky mechanism considers that electrons with sufficient energy will be able to overcome the potential barrier $\phi_{0}$, where the required energy source is associated with an increase in temperature T or an external electric field E. Under a high electric field, the decrease of the potential barrier is related to an image force, due to an electrostatic field created by the electron that has left the electrode, and the charge induced by the opposite sign in the electrode [12]. This attraction produces a gradual change of the potential barrier and is considered the potential energy of the electron. Therefore, the current density J is given by Equation (1). It is important to remark that in this equation a trap-free material was assumed, without physical or chemical defects.

(1) $$J={A^{*}T}^{2}\exp\left[-\frac{\left(\phi_{0}-\beta_{S}E^{\frac{1}{2}}\right)}{\mathrm{kT}}\right]$$

where $A^{*}$ is Richardson’s constant. If the electric charge carrier is an electron, this constant has a value of 120 A/cm^2^·K^2^. $\beta_{S}$ is the Schottky constant, T is the absolute temperature in K, m is the mass of the electron, q is the charge of the electron, k is the Boltzmann constant and h is the Planck constant.

When the electron cannot overcome the potential barrier, there is the possibility that, due to the wave–particle duality, it can cross it, and thus inject from the electrode into the polymer. This phenomenon, known as the tunneling effect or Fowler–Nordheim mechanism, is approached by the principles of quantum mechanics [20]. The deduction of the equation that governs this mechanism considers a triangular potential barrier, which is created due to the potential energy of the electron. Equation (2) defines the current density, which is independent of temperature. This phenomenon typically occurs in insulators and dielectrics at high electric fields in the order of 10^8^–10^9^ V/m.

(2) $$J=\frac{q^{3}E^{2}}{8\pi h\phi_{0}}\exp\left(-\frac{{8\pi \sqrt{2m}\phi_{0}}^{\frac{3}{2}}}{3\mathrm{hqE}}\right)$$

Under low electric fields, Ohm’s law can describe the conduction mechanisms in the bulk polymer. At high electric fields, the voltage–current curves deviate from a linear relationship. If the electrons are injected into the polymer and if the relative dielectric permittivity, $\varepsilon_{r}$, is high (greater than 10), a high concentration of electrical charge is generated within the material. Consequently, more electrical charge is injected into the polymer than can be released. It is generally assumed that the injected electrical charge is evenly distributed spatially throughout the material; however, various structural phenomena prevent the charges from being distributed symmetrically. In this context, the space charge-limited current (SCLC) is a measure of the rate of injection and extraction of electric charge carriers into the bulk material [12,15,21]. This electrical behavior depends on the concentration of charge carriers, the type of electrical charge, its mobility, the nature of the electrodes and the trapping characteristics. Equation (3), also known as the Mott–Gurney equation, gives the current density. It considers a polymer without traps and thermal generation of charge carriers. Having an ohmic contact, which involves injecting a single type of charge carriers, is also desirable.

(3) $$J=\frac{9}{8}\varepsilon_{0}\varepsilon_{r}\mu \frac{E^{2}}{d}$$

In Equation (3), $\varepsilon_{r}$ is the relative permittivity of the material, $\varepsilon_{0}$ is the dielectric permittivity of the vacuum, d is the thickness of the polymer film, and μ is the mobility of the electric charge carriers. It is important to note that J is proportional to the square of the electric field, and inversely proportional to the thickness. On the other hand, for the case of a polymeric material in which the single energy level trap density is assumed, a modified expression, given by Equation (4), is obtained for J. Equation (4) considers the factor θ, denoting the proportion between free and trapped carriers.

(4) $$J=\frac{9}{8}\varepsilon_{0}\varepsilon_{r}\mu \;\theta \frac{E^{2}}{d}$$

Another bulk effect is the Poole–Frenkel mechanism, analogous to the Schottky mechanism. Considering an energy potential well associated with a trap, the Poole–Frenkel effect is the result of the reduction of the trapping energy under the combined effect of the Coulomb potential and the electric potential associated with the external electric field. In polymeric materials in thin film form, the trapping centers are considered fixed, therefore, conduction is carried out only by electrons that have crossed the lowered potential barrier. The expression of the Poole–Frenkel current density is given by Equation (5), where $J_{0}$ is a pre-exponential factor, and $\beta_{\mathrm{PF}}$ is the Poole–Frenkel constant.

(5) $$J=J_{0}\exp\left[-\frac{\left(\phi_{0}-\beta_{\mathrm{PF}}E^{\frac{1}{2}}\right)}{\mathrm{kT}}\right]$$

Ions exist in large amounts in polymers and can originate from impurities, depending on the synthesis and manufacturing procedures. These can also be produced by ionization processes, such as radiation absorption, material breakdown, and contaminant absorption. The motion of ions results in mass transport, and their size is too large compared with the size of the electrons, hence ionic mobility is several orders of magnitude lower than electron mobility. In this case, the physical transport mechanism occurs through a series of jumps, where λ represents the average distance between jumps over the potential barriers, allowing the ions to move from one place to another through the bulk [22]. The complex structure of the polymer creates potential barriers and can be modified by applying an electric field or by increasing temperature. An expression that defines the current density for the ionic conduction mechanism is Equation (6), following the hypothesis of uniformly distributed traps.

(6) J=J0sinhqλE2kT

## 3. Materials and Methods

Samples were manufactured in the form of a thin film from solutions of ethyl cellulose EC with an average molecular weight of 135,000 g/mol (Dow Chemicals, Rochester, NY, USA) in tetrahydrofuran (THF, 99%, Sigma-Aldrich, St. Louis, MO, USA) as a solvent. A concentration of 10% by weight was used, ensuring an appropriate film manufacture [2]. To guarantee the dissolution process, the EC–THF mixture was stirred at 700 rpm for 1 h, at 313 K. The solutions obtained were subjected to a tape casting process (this process controls the film thickness) on a polytetrafluoroethylene (PTFE) surface, to separate the solvent by natural convection, at room temperature for 24 h. The EC films obtained were stored in a desiccator for later use. The thickness of the obtained films was measured with a micrometer, obtaining a value of ~20 ± 2 μm.

The viscoelastic response of the EC was analyzed by DMA, using a Perkin Elmer DMA8000 (Waltham, MA, USA), under isochronous conditions (0.1, 1 and 10 Hz), in tension mode, with a heating ramp of 2 K/min, from 298 to 453 K [8]. EC was analyzed by X-ray diffraction (XRD) to determine the presence of amorphous or crystalline phases. The XRD analysis was undertaken using a PANalytical diffractometer Empyrean model, with CuKα radiation at the wavelength of λ =0.154 nm, over the range from 5 to 80° (2θ); the sample was previously pulverized to perform the XRD analysis.

The EC films were subjected to a constant direct current (DC) voltage for the electrical characterization and measurement of transient currents. The experimental device consists of two flat and parallel copper electrodes of 25 mm diameter. The polymer sample was placed between the electrodes to obtain a capacitor configuration. The external electrical circuit of the configuration was connected to a Keithley 6517B electrometer (Beaverton, OR, USA), which has a resolution of 10^−14^ A. The electrical characterization was carried out in two stages. The measurements of I(t) were recorded at different step voltages for a time of 1000 s (polarization current), and the applied voltage was then reduced to zero for a time of 1000 s (depolarization current). After this, the previous protocol was repeated, but applying a step voltage of greater magnitude. In total, curves I(t) were recorded for DC voltages of 100, 200, 300, 400 and 500 V.

This experimental procedure was carried out at room temperature (298 K) and constant pressure, where the electrode–polymer set was placed in a vacuum chamber, with the aim to reduce the effect of water molecules on the measurements. The experimental data were used to identify the conduction mechanisms. Because the physical models (Section 2) relate an electric current density (J=I/A) with an electric field (E=V/d), the observed I–V were transformed into data pairs J–E. For this purpose, the geometry of the sample analyzed was considered (area A and thickness d).

Additionally, alternating current (AC) conductivity measurements were carried out using the electrode–polymer set. An Agilent E4980A electrometer (Santa Clara, CA, USA) was used in a frequency range from 20 Hz to 2 MHz. The applied voltage follows an oscillating signal between −1 and 1 V; the measurements were performed above the glass transition temperature, from 403 K to 473 K.

## 4. Results and Discussion

### 4.1. Dynamic Mechanical Analysis

**Figure 1:**
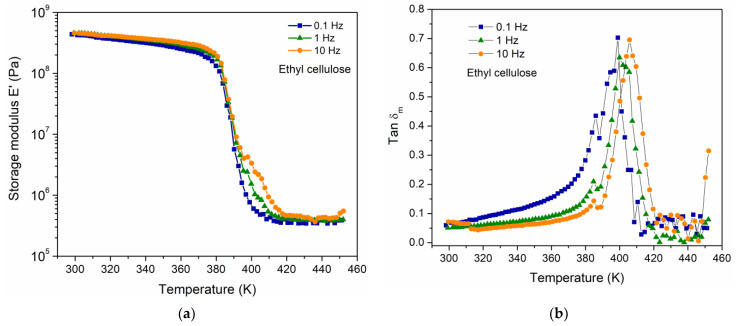
(**a**) E′ and (**b**) tan δ_m_, as a function of temperature and three different frequencies for EC.

The viscoelastic response of the EC was carried out using DMA, to determine the mechanical manifestation of the relaxation phenomena. The DMA results are presented in Figure 1a,b and correspond to the temperature dependence of storage modulus E′ and tan δ_m_, respectively, for three frequencies of the applied mechanical stimulus (0.1, 1 and 10 Hz). Figure 1b shows that the Tg (defined as the peak position in tan δ_m_) is a function of frequency as expected in viscoelastic materials [2,8]. The magnitude of the Tg for EC at the frequency of 1 Hz corresponds to 402 K, which is comparable to that reported by Davidovich-Pinhas et al. (Tg = 403 K) [23]. Additionally, through a DSC measurement for the EC at a heating rate of 5 K/min, a value of 397 K was obtained for the Tg. A thermal event associated with the fusion of crystals in the polymer was also observed, at a temperature of 451 K, suggesting a semicrystalline behavior in the polymer.

### 4.2. X-ray Difraction Analysis

**Figure 2:**
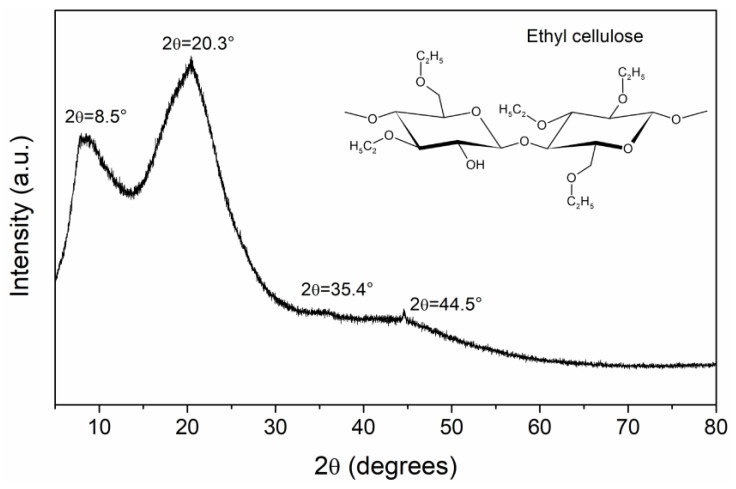
XRD results for EC powder. In the inset of the figure the chemical structure of EC is presented.

X-ray diffraction pattern for EC powder is presented in Figure 2. This measurement is important because the electrical properties of polymers are very sensitive to structure and morphology [15]. The diffraction pattern shows two scattered peaks; this means that EC has a specific order within the amorphous background (short-range order). In the literature it is reported that EC presents an intimate mixture of amorphous and crystalline phases [1,23]. The first peak is centered at 2θ =8.5° (d =10.39 Å), which is associated with the distance between the ordered structures of the polymer, and the second peak at 2θ =20.3° (d =4.37 Å) gives information about the interchain distance. Another two weak peaks are detected, at 2θ =35.4° (d =2.53 Å) and 2θ =44.5° (d =2.03 Å). These distances, d, were calculated using Bragg’s equation (2dsin⁡θ=λ). The degree of crystallinity was calculated considering the area of crystalline and amorphous peaks, obtaining a value of 53.77%. The crystallite size was calculated using the Scherrer equation, obtaining a value of 10.08 Å, the order of magnitude of the cellulose dimer length [23]. From an electrical point of view, amorphous regions function as traps with different energy levels, generating a complex electrical behavior in the polymer.

### 4.3. Transient Currents

**Figure 3:**
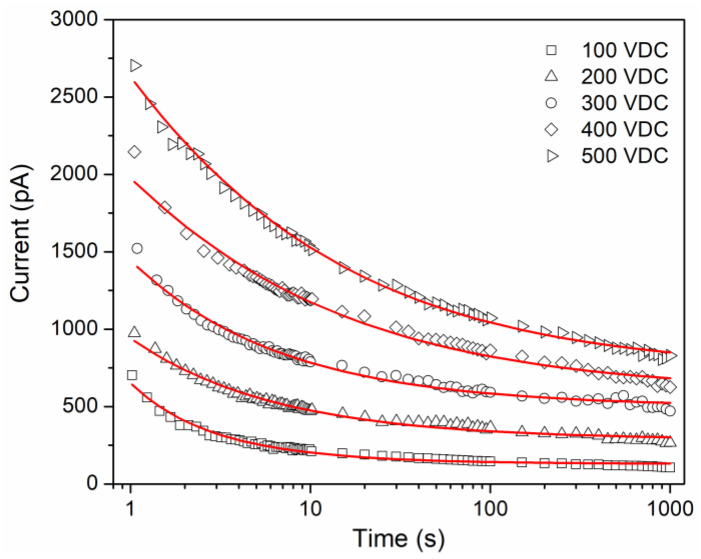
Comparison between isothermal transient currents for EC (symbols) and continuous lines represents the best fitting result.

Figure 3 shows the isothermal curves $I\left(t\right)$ obtained for the applied DC voltages. The experimental curves describe electrical currents in an order of magnitude of the pA, and increase globally as the applied DC voltage increases, but each curve shows a decrease in current as time increases. The shape of the curves does not seem to be affected significantly by the applied voltage; this behavior can be considered as a qualitative indicator of the movements induced by electric charge carriers because it is a relaxation phenomenon. The transient currents are analogous to stress relaxation experiments, within the linear viscoelastic regime. In a polymer, under an external electric field, the formation of new electric dipoles is induced. These dipoles, as well as the existing dipoles, orient in the direction of the applied field, with a rate that depends mainly on the viscoelastic nature of the polymer [11,24]. This dipole orientation process occurs macroscopically as a polarization current, which is a function of the number of dipoles and the rate with which they are oriented. When the electrical stimulus is removed, an electric current of the opposite sign is obtained (depolarization current), with a tendency to a zero value, which is related to the random redistribution of the electric dipoles caused by thermal agitation, which in turn tends to return to its initial conformational state. One electrical charge can be stored (capacitive response), and the other is dissipated as electric current (resistive response). Considering the chemical groups that make up the molecular structure of the EC, the permanent dipoles hydroxyl, pyranose ring, ethoxy and methoxy [11], dipolar orientation is established as a conduction mechanism that contributes to the shape of the transient currents $I\left(t\right)$.

In the literature it has been established that the transient currents follow an empirical function, the well-known Curie-von Schweidler law, $I(t)=At^{-n}$. However, this only considers a unique relaxation process or conduction mechanism, ignoring a steady-state conduction. As a first approximation, the curves $I\left(t\right)$ for several insulators have been modeled empirically considering a negative exponential function, obtained from an equivalent resistor–capacitor circuit [25,26]. The resistor represents a conductive behavior in the material, and the capacitor represents a dielectric behavior. The classical expression for this circuit is $I\left(t\right)=I_{0}\exp(-t/\tau )$, where $I_{0}$ is a pre-exponential factor, τ=RC is the relaxation time in seconds, R is the resistance in Ohms and C is the capacitance in Farads. According to this model, and if only the orientation of permanent or induced electric dipoles is presented, it can be concluded that, as the applied DC voltage increases, the global increase of the curves $I\left(t\right)$ is a direct consequence of many electric dipoles that are oriented when the electric field increases. To obtain the best fit to experimental data, using a generalized model of resistors and capacitors is an alternative, with a higher number of RC elements, but an increase in the number of parameters. The poor description obtained when we use exponential function to fit experimental data can be improved using a Mittag–Leffler function [27,28]. The Mittag–Leffler function $E_{\alpha }$ of one parameter α is defined as Equation (7):

(7) $$E_{\alpha }\left(z\right)=\sum_{k=0}^{\infty }\frac{z^{k}}{\Gamma \left(\alpha k+1\right)}$$

where $\Gamma \left(\cdot \right)$ is the gamma function, α > 0 and z∈C. If α =1, we obtain the exponential function exp⁡(z). In this sense, Mittag–Leffler function is considered a generalization of the exponential function. A fractional differential equation for an RC circuit is obtained using the fractional calculus approach [29], Equation (8), which relates the voltage V(t) and the current $I\left(t\right)$. The fractional derivatives are described by Caputo derivative definition.

(8) $$C\tau^{\alpha -1}\frac{d^{\alpha }V(t)}{dt^{\alpha }}=\tau^{\alpha }\frac{d^{\alpha }I\left(t\right)}{dt^{\alpha }}+I\left(t\right)$$

The solution for Equation (8) is obtained using the Laplace transform, considering $V\left(t\right)=V_{0}u(t)$, where u(t) is the unit step function, and an initial condition $I\left(0\right)=0$:

(9) $$I\left(t\right)=I_{0}\cdot E_{\alpha }\left[-{\left(\frac{t}{\tau }\right)}^{\alpha }\right]$$

where α is a fractional order that takes values between 0 and 1. Equation (9) is useful for fitting experimental data considering the polarization current and relaxation phenomena. If α = 1, we recover the classical expression for transient current (exponential function). The Mittag–Leffler function presents two interesting asymptotic approximations, at small times (t→0), $E_{\alpha }\left({-z}^{\alpha }\right)\sim \;\exp[{-z}^{\alpha }/\Gamma (1+\alpha )]$, and at large times (t→∞), $E_{\alpha }\left({-z}^{\alpha }\right)\sim z^{-\alpha }/\Gamma (1-\alpha )$. This means that $E_{\alpha }\left(\cdot \right)$ interpolates between the stretched exponential function (Kohlrausch–Williams–Watts function) and a negative power-law (Curie-von Schweidler law) [28,30].

According to the nature of the transient currents, in this work the total current is expressed as the sum of the polarization current $I_{p}$ and the conduction current $I_{c}$.

(10) $$I\left(t\right)=I_{p}+I_{c}=I_{0}\cdot E_{\alpha }\left[-{\left(\frac{t}{\tau }\right)}^{\alpha }\right]+I_{c}$$

Figure 3 compares the experimental data and the modeling, which is considered acceptable. The fitting procedure was performed in MATLAB using a routine proposed by Podlubny [31]. Table 1 shows the values of the parameters involved in Equation (10). $I_{c}$ presents an increase as the voltage or electric field increases, which in other polymer systems is observed [15,32]. For $I_{0}$, a relationship as a function of voltage is not presented, which is due to the manifestation of different conduction mechanisms at very short times. The obtained values for the fractional order α are between 0 and 1. The fractional order α can be interpreted as a measure of the rate of energy dissipation in the polymeric material. When the voltage increases, the fractional order decreases. This can be associated with a greater storage of electric charge, mainly due to the number of electric dipoles that are oriented under the electric field. However, dissipative phenomena are still present, coupled with the different conduction mechanisms that are manifested. In addition, the relative average absolute deviations (AAD) present lower values as the voltage increases, which refers to a better fit compared with the classic exponential function.

### 4.4. Analysis of Conduction Mechanisms

**Figure 4:**
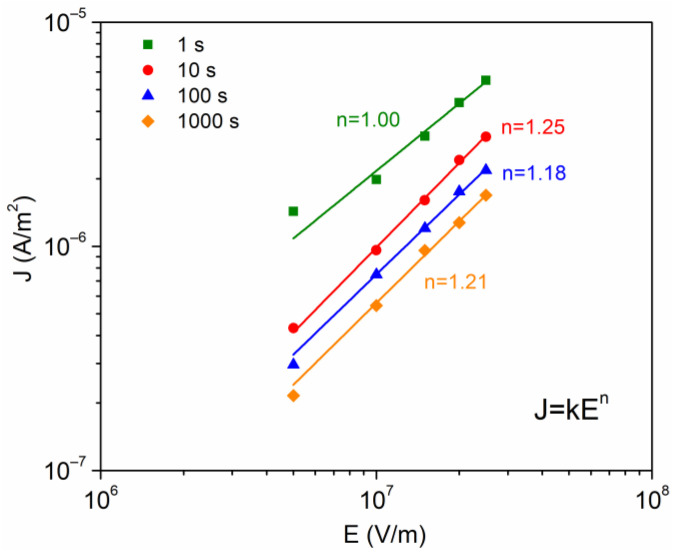
Current density as a function of the electric field, at different times of analysis.

The transient currents $I\left(t\right)$ of Figure 3 were analyzed using the physical models summarized in Section 2. From these curves $I\left(t\right)$ for each of the applied voltages, a data set J–E was constructed for several values of time t (1, 10, 100 and 1000 s), and Figure 4 was constructed. The experimental data were adjusted to the expression $J=kE^{n}$, which allows one to characterize whether or not there is an ohmic contact between the electrodes and the sample under analysis. Only for t = 1 s is the ohmic contact satisfactory because n = 1, and for the others times the values of index n present a slight deviation from unity. The DC conductivity of the EC was calculated at t = 1 s, σ = 2.5 × 10^−13^ S/m, which is a function of the density of electric charge carriers present in the EC, and its respective mobility; this calculated value is in the order of magnitude reported for other polysaccharides [11,33].

**Figure 5:**
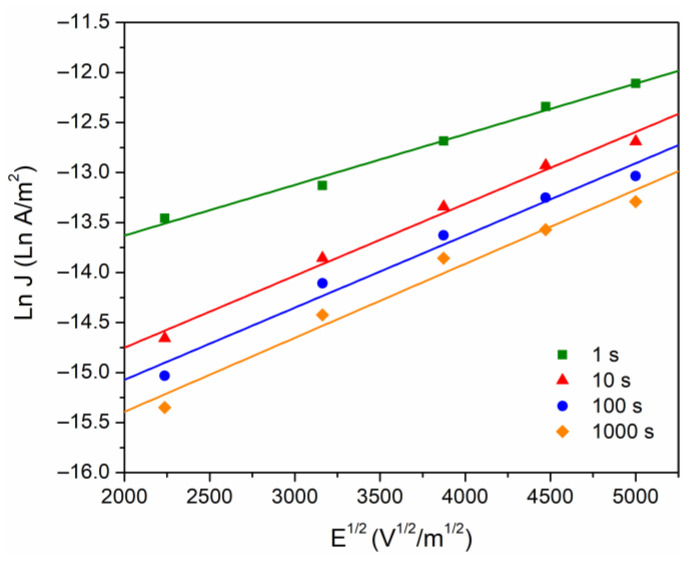
Ln J versus E^1/2^, at different times of analysis.

In the literature it is accepted that the presence of space charge modifies the electric field at the electrode–polymer interface, where there is a competition between the polarization charges and the charges injected into the polymer. In this sense, we proceed with the analysis of the conduction currents that can be controlled by the electrode–polymer interface. The two main injection mechanisms will be considered: Fowler–Nordheim (Equation (2)) and Schottky (Equation (1)). The Fowler–Nordheim effect (quantum mechanical phenomenon) controls the current if the plot $\ln\left(J/E^{2}\right)$ versus $\left(1/E\right)$ is linear and with a negative slope. This condition is not satisfied in our case, because this phenomenon requires thin insulating barriers that can only be achieved at high electric fields (∼10^9^ V/m). Concerning the Schottky model, this is generated by the reduction of potential barrier in the interface due principally to external electric field, which implies that electrons can be injected into the polymer. The data were calculated from Figure 4, taking $\ln\left(J\right)$ versus $E^{1/2}$, as given by Figure 5.

Furthermore, Poole–Frenkel (Equation (5)) is also considered with respect to bulk conduction mechanisms. According to the Poole–Frenkel effect, the conduction current is induced by a decrease of the potential barrier of the traps, under the combined effect of the Coulomb potential and the electric potential originated by the external electric field. The equations for Schottky and Poole–Frenkel are similar, and in consequence it is not easy to identify the mechanisms responsible for the conduction (if the electrons are compensated, $\beta_{\mathrm{PF}}=2\beta_{S}$). From the linear fit for data of the Figure 5, the slopes were calculated to obtain $\beta_{\mathrm{PF}}$ and $\beta_{S}$, which are presented in the Table 2. Miranda-Valdez et al. [34] report a relative permittivity of $\varepsilon_{r}$ = 5 at 1 MHz for EC, with this value used to calculate theoretical $\beta_{\mathrm{theo}}$= 2.71 × 10^−24^ J m^1/2^/V^1/2^. This result agrees with the values obtained for the Schottky mechanism (Table 2), suggesting that, at voltages greater than 100 V, there is a probability that charge carriers are injected into the polymer through the interface. The height of the energy barrier was ~1.07 eV, which agrees with that typically observed in electrode–polymer-electrode systems, from 1–1.5 eV [15,32].

**Figure 6:**
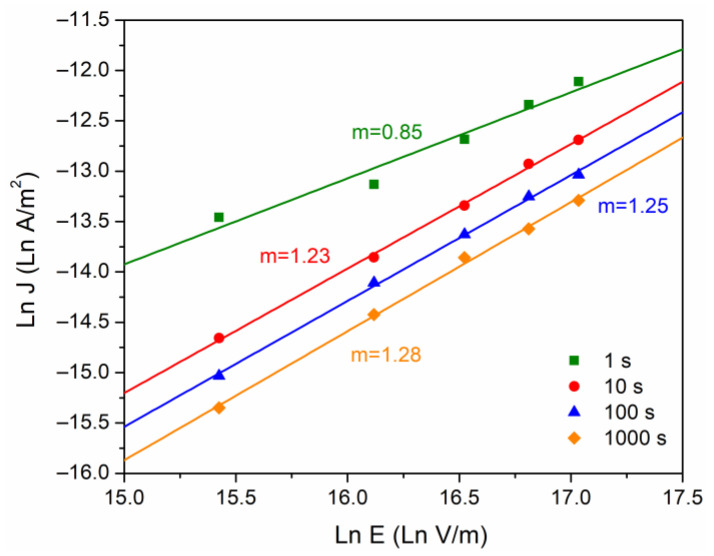
Ln J versus Ln E, at different times of analysis.

For the case of the conduction currents controlled by the polymer bulk, the SCLC model was also considered (Equation (4)). Figure 6 was also constructed from Figure 4, taking Ln(J) vs Ln(E). The purpose of this graph is to observe a slope change (denoted as “m”). With m = 1 it is said that the conduction mechanism follows an ohmic behavior, and if m = 2, the mechanism is associated with the presence of space charge [21]. The calculated values for m vary from 0.84 to 1.28, as time increases. If we consider in this case a material with a trapping density, which is a consequence of the complex semicrystalline structure, there is a tendency to accumulate electric charge.

**Figure 7:**
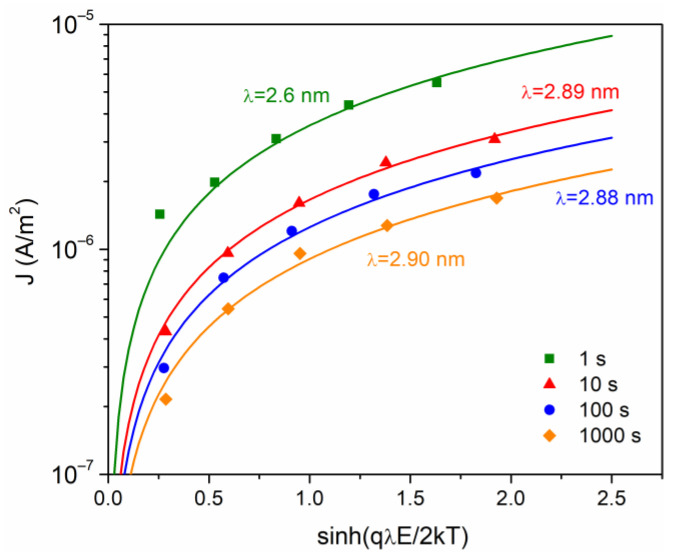
Current density versus sinh(qλE/2 kT), at different times of analysis.

Additional analysis of conduction mechanism is very important considering the current in terms of mobile ions, defect sites and a carrier hopping process. A model initially developed for ionic conduction (Equation (6)) leads to a density current proportional to ~$\sin h\left(q\lambda E/2\mathrm{kT}\right)$, where λ is the jump distance between potential wells. To obtain the fit with the data, the method used by Guillermin et al. was used [32]. The values obtained are presented in Figure 7 and are consistent with others reported for different polymers [22,35]. At t = 1 s, a value of λ = 2.6 nm is calculated, while for the other times values of λ~2.8–2.9 nm were calculated. The tendency for the jump distance to remain principally unchanged in the glassy state is related to the small change in chain arrangement caused by the frozen micro-Brownian motion [22].

Based in these models, it is established that the isothermal curves I(t) obtained for the EC are the result of the contribution of electron injection, dipolar orientation, and ionic conduction. On the other hand, there is no evidence of SCLC under the conditions analyzed. This is not enough to establish that the injected electrons have not restricted their mobility due to the traps (shallow or deep) in the bulk of the polymer, which would produce an accumulation of electrical charge. However, the presence of space charge can be stimulated by a considerable increase in temperature, or the injection of electrons at high electric fields.

### 4.5. AC Electrical Conductivity

**Figure 8:**
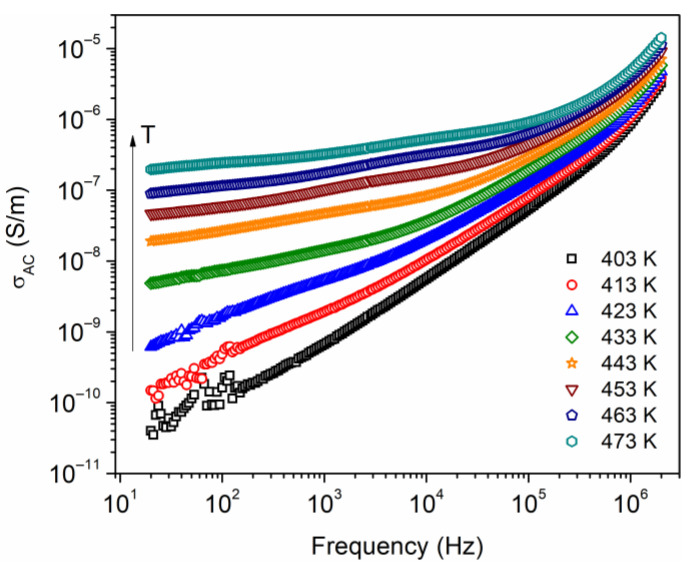
AC conductivity as a function of the frequency, at different temperatures.

AC electrical conductivity, $\sigma_{\mathrm{AC}}$, measurements at temperatures above glass transition temperature (Tg = 397 K from DSC) were carried out. Figure 8 shows $\sigma_{\mathrm{AC}}$ as a function of frequency, from 20 Hz to 2 MHz, and at temperatures from 403 to 473 K. At low frequencies, an increase in $\sigma_{\mathrm{AC}}$ is denoted as the temperature increases; this suggests that the $\sigma_{\mathrm{AC}}$ is a thermally activated process and can be associated with an increase of the mobility of charge carriers. This is favored due to the increment in free volume and an increase in segmental motions of polymer chains. A nonlinear dependence on conductivity with frequency is observed, where this dependence is presumed to be due to hopping transport between the localized states. Additionally, it is possible that electrode polarization is present due to a charge accumulation near the electrodes, which is observed at low frequencies and in the temperature range of 403–433 K. Jonscher proposed an equation relating AC conductivity as a function of frequency, $\sigma_{\mathrm{AC}}=A\omega^{s}$, which follows the form of a power law [36], where A is a constant and the exponent s takes values typically between 0 and 1. This behavior has been observed in disordered solids and amorphous polymers at high frequencies [37,38]. The equation did not fit adequately to the data in Figure 8 in the analyzed frequency spectrum. A possible explanation for this is the presence of various conduction mechanisms, such as the Maxwell–Wagner–Sillars (MWS) interfacial relaxation, which originates from the heterogeneous structure of the semicrystalline polymer [39].

**Figure 9:**
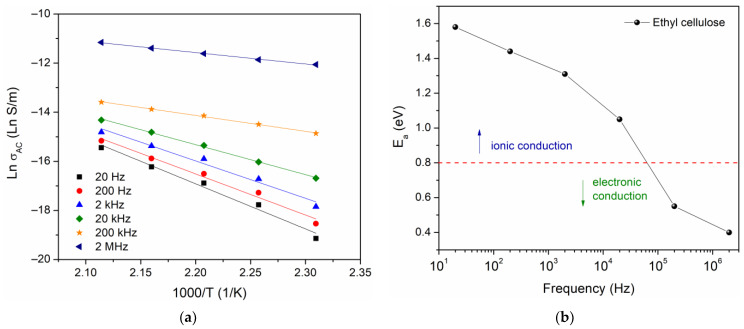
(**a**) Ln $\sigma_{\mathrm{AC}}$ versus 1000/T and (**b**) activation energy as a function of frequency.

From the data in Figure 8, an Arrhenius equation $\sigma_{\mathrm{AC}}=\sigma_{0}\exp\left(-E_{a}/\mathrm{kT}\right)$ was used to calculate the activation energy $E_{a}$, where $\sigma_{0}$ is a pre-exponential factor, k is Boltzmann’s constant, and T is the absolute temperature. The Ln $\sigma_{\mathrm{AC}}$ vs. 1000/T has been plotted, for which the activation energy has been calculated for six different frequency values, shown in Figure 9a. The results are presented in Figure 9b, where one can observe a decrease in the activation energy as the frequency increases.

In the literature, the approach proposed by Jonscher and Ansari has been used for the identification of conduction mechanisms. For this approach, $E_{a}$ being higher than 0.8 eV indicates a dominant ionic conduction, whereas $E_{a}$ being less than 0.8 eV indicates that the dominant conduction mechanism is through the motion of electrons [40]. The value obtained in this work $E_{a}$ = 1.6 eV, at low frequency, suggests a conduction mechanism governed by the motion of the macromolecular chains of ethyl cellulose. However, the presence of space charge and the phenomenon of interfacial relaxation should not be ruled out.

## 5. Conclusions

This study has confirmed the semicrystalline structure of EC, which consists of an intimate mixture of amorphous and crystalline phases. The XRD tests revealed a crystal size of about 10 Å and a degree of crystallinity of 54%. From DMA it was observed that the EC presents a main mechanical relaxation at 402 K, which is related to cooperative molecular movements of the glass transition phenomenon. The transient currents presented an exponential decay as a function of time. The comparison between experimental data and the solution from the fractional differential equation for the RC circuit shows good agreement. The fractional order takes values between 0 and 1, and it has been found that this parameter lowers as the voltage increases, indicating increased electric charge storage.

The interpretation of the voltage–current data has given a positive result to the Schottky mechanism, with injection of charge carriers through the electrode–polymer interface at voltages greater than 100 V. At the level of the bulk polymer, the electric current generated is the result of the orientation of the electric dipoles and ionic-hopping process. Under the voltage and temperature conditions employed in this work, space charge was not detected. AC conductivity measurements yield an activation energy of 1.6 eV, at high temperatures and low frequencies, indicating that electric conduction is governed by the mobility of EC chain segments.

## Figures and Tables

**Table 1:** Fit results from experimental data.

Parameter	100 V	200 V	300 V	400 V	500 V
I_c_ (pA)	129.61	286.06	497.81	591.51	726.03
I_0_ (pA)	6289	11,532	16,290	7055	9120
τ (s)	0.1519	0.0159	0.0135	0.0707	0.0866
α (-)	0.75	0.53	0.52	0.41	0.41
AAD (%)	6.41	3.63	2.49	2.67	1.88

**Table 2:** Fit results for the Schottky and Poole–Frenkel constants from experimental data.

Parameter	1 s	10 s	100 s	1000 s
$\beta_{S}$ (J m^1/2^/V^1/2^)	2.08 × 10^−24^	2.96 × 10^−24^	2.97 × 10^−24^	3.04 × 10^−24^
$\beta_{\mathrm{PF}}$ (J m^1/2^/V^1/2^)	4.17 × 10^−24^	5.91 × 10^−24^	5.94 × 10^−24^	6.09 × 10^−24^

## Data Availability

The data generated during the current study are available from the corresponding author upon reasonable request.
